# Health Economic Considerations for the Implementation of Artificial Intelligence‐Enabled Diabetic Retinopathy Screening: A Review

**DOI:** 10.1111/ceo.70016

**Published:** 2025-11-03

**Authors:** James Leigh, Jocelyn Drinkwater, Angus Turner, Elizabeth‐Ann Schroeder

**Affiliations:** ^1^ Nuffield Department of Primary Care Health Sciences University of Oxford UK; ^2^ Lions Eye Institute, Lions Outback Vision Nedlands Western Australia Australia; ^3^ Centre for Ophthalmology and Visual Science The University of Western Australia Crawley Australia

**Keywords:** cost‐effectiveness, deep learning, implementation science, machine learning, retinal diseases

## Abstract

Artificial intelligence (AI) has comparable accuracy to ophthalmologists for diabetic retinopathy (DR) screening, yet its cost‐effectiveness is crucial for implementation. Our review of 18 health economic analyses of AI versus manual grading for DR found significant methodological variation, with cost‐utility analysis and Markov modelling being the commonest evaluation and modelling approaches, respectively. We identified three key considerations when appraising health economic analyses of AI‐enabled DR screening: the importance of contextualised parameters including subgroup analysis, real‐world data on adherence to ophthalmology follow‐up, and the trade‐off between diagnostic accuracy and cost‐effectiveness. 39% of studies followed standardised reporting guidelines, and most did not consider improved follow‐up after AI screening, potentially underestimating its economic value. Future evaluations should incorporate contextualised parameters, including adherence and regional data, and recognise that the most accurate diagnostic screening may not reflect the most cost‐effective. Studies should follow updated reporting guidelines such as CHEERS‐AI or PICOTS‐ComTeC to improve methodological transparency.

## 1 Introduction

Diabetic retinopathy (DR) is one of the leading causes of blindness worldwide, affecting approximately one in three people with diabetes [1]. An estimated 828 million people live with diabetes globally [2], and its retinal consequences are a significant public health concern, manifesting as a three‐fold increase in global prevalence over the past 20 years [3]. DR imposes a substantial burden on people's quality of life and the economic costs to healthcare systems and societies are immense [1, 4]. Screening programs and treatment for DR are considered highly cost‐effective in the prevention of vision loss from diabetes [5], however despite their widespread implementation around the world [6] the rates of screening are sub‐optimal [7].

The economic burden of DR is multifaceted and substantial. In the United States, the Centre for Disease Control estimates the total cost of retinal disorders in patients with diabetes to be $4.1 billion annually, corresponding to $3640 per person [4] and $800 million in Medicare payments [8]. Similarly, in Australia, indirect costs associated with diabetic macular oedema alone are estimated at $2.07 billion [9]. Direct medical costs primarily stem from treating advanced disease through interventions such as pan‐retinal photocoagulation or intravitreal injections performed by specialists [10]. Additional direct non‐medical costs include equipment purchases and transportation expenses incurred by patients and families, while indirect societal costs encompass productivity losses, caregiving needs, and lost income. Since advanced disease drives these major costs, early detection through effective screening is essential to prevent irreversible vision loss and its associated economic burden [11].

Artificial intelligence (AI) algorithms have emerged as potentially effective tools for DR screening, with several systems receiving regulatory approval, either as decision support tools, for example, Google's ARDA [12], or as autonomous tools, for example, EyeArt [13] and IDx‐DR [14]. Generally speaking, AI‐enabled DR screening occurs once a fundoscopic image is obtained by a trained technician and uploaded to a cloud‐based server, usually without the need for pupil dilation. Once uploaded, the algorithm grades the patient's retinal image instead of a trained human grader, and determines whether a referral is indicated [15]. These algorithms have demonstrated comparable or superior diagnostic accuracy to expert clinicians in internal and external validation studies [12, 13, 14].

Despite growing interest in implementing AI‐enabled DR screening, particularly in low‐resource settings, the current state of knowledge regarding their economic implications remains fragmented. Value proposition is recognised in implementation science as a critical barrier or enabler to the adoption, scale‐up and spread of digital health interventions such as AI [16]. Furthermore, while several health economic evaluations have been published, there exists considerable uncertainty about how methodological differences might affect the quality of studies and the cost‐effectiveness of these technologies across different healthcare contexts.

This review aims to identify and synthesise the existing literature regarding health economic analyses of AI‐enabled DR screening with consideration of its real‐world implementation. Specifically, we seek to characterise the methodological approaches used for health economic analyses of AI‐enabled DR screening; assess how context‐specific parameters and patient adherence impact cost‐effectiveness outcomes; and evaluate the use of established reporting guidelines such as the Consolidated Health Economic Evaluation Reporting Standards (CHEERS) checklist among included studies. Our review was conducted with reference to the Cochrane Library Guidelines [17] and reported using an adapted version of the Preferred Reporting Items for Systematic Reviews and Meta‐Analysis (PRISMA) guidelines [18].

## 2 Methods

### 2.1 Study Design

A systematic search strategy was conducted using Embase, MEDLINE and Scopus databases from inception to January 30, 2025. We utilised these databases to capture economic evaluations alongside clinical and population health interventions, cross‐disciplinary health economic evaluations and European health economic evaluations, respectively. The population of interest included people with diabetes at risk of, or with known, DR who require routine screening as per the guidelines in their respective countries or healthcare settings. The intervention was AI‐enabled DR screening, including machine learning or deep learning algorithms that have been developed and validated to detect DR, compared with manual grading. In this review, AI‐enabled DR screening includes both semi‐automated and automated screening. Semi‐automated AI screening refers to an initial image analysis by an AI algorithm with subsequent review by a manual grader only for positive results. If confirmed positive, patients are subsequently referred on for specialist review. Automated AI screening refers to DR screening whereby the AI is entirely responsible for diagnosing and referring patients to a specialist, without any confirmatory review by a manual grader. Our outcomes related to cost‐effectiveness metrics, parameter selection including data on adherence to follow‐up, and use of reporting guidelines. Table 1 outlines our expanded Population, Intervention, Comparator and Outcome (PICO) framework underpinning the research question. We used a narrative approach to synthesise the literature and present our findings.

**TABLE 1:** Expanded PICO framework applied to narrative review research question.

P	POPULATION	Patients with type 1 or type 2 diabetes at risk of, or with, DR requiring routine screening
I	INTERVENTION	AI‐enabled grading for DR screening
C	COMPARATOR	Manual grading for DR screening, in‐person or via telehealth
O	OUTCOME	Any measure of effectiveness which may include clinical indicators – Utility measured as quality‐adjusted life years (QALYs) – Appropriate outcome – Referable cases detected
	Costs	All costs from a healthcare system and societal perspective
	Economic evaluation	Cost‐effectiveness of AI‐enabled DR screening compared with manual grading
	Special consideration	Context‐specific parameter selection; consideration of adherence rates to follow‐up; utilisation of standardised reporting guidelines

### 2.2 Search Strategy

Our search strategies across each database were developed in collaboration with an Information Specialist. We used the following search in MEDLINE to demonstrate our strategy: (Artificial Intelligence/ or Neural Networks, Computer/ or exp. Machine Learning/ or Image Processing, Computer‐Assisted/ or (Automated or “autonomous AI” or “computer based analysis” or “convolutional neural network”).ti,ab,kw.) and ((Ophthalmology/ or Diabetic Retinopathy/ or (“Diabetic eye*” or “diabetic retinopathy” or “retina” or “retinal” or “retinopathy”).ti,ab,kw.) and (Cost–Benefit Analysis/ or Cost‐Effectiveness Analysis/ or Cost* or “health economic*”).ti,ab,kw.). A full description of our search strategy translated across MEDLINE, Embase, and Scopus databases is available in Table S1. Forward and backward citation checks were performed on articles included for final analysis to identify any additional articles for analysis based on eligibility criteria.

### 2.3 Inclusion Criteria

Publications included for review had to be primary research; had to include a health economic analysis of patients undergoing DR screening using artificial intelligence; and be written in English.

### 2.4 Exclusion Criteria

Publications excluded from review were non‐original research articles such as case reports, commentaries, letters to the editor; review articles; articles without full‐text availability; and research protocols. Non‐original research that is relevant to the topic was considered for inclusion within our discussion to broaden perspectives regarding health economic evaluations of AI‐enabled DR screening. Table S2 provides an overview of our inclusion and exclusion criteria.

### 2.5 Quality Assessment and Risk of Bias

Risk of bias for included studies was assessed using the Risk of Bias in Non‐randomised Studies—of Interventions (ROBINS‐I). Quality assessments including study limitations were performed using the National Institute for Health and Care Excellence (NICE) appraisal checklist for economic evaluations, modified to focus on study limitations to supplement our risk of bias assessment.

## 3 Results

### 3.1 Summary of Included Studies

**FIGURE 1:**
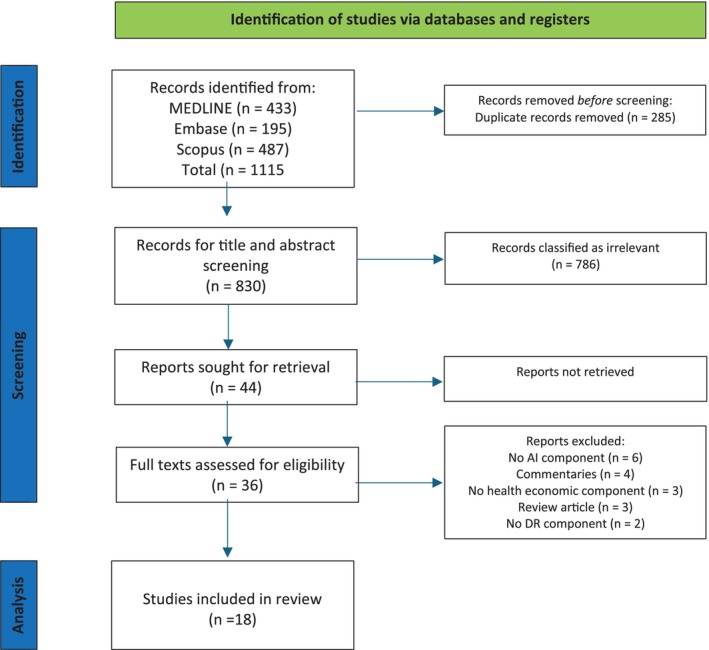
PRISMA diagram showing literature review methodology.

A total of 1115 articles were identified from our literature search of MEDLINE, Embase and Scopus. After the removal of duplicates there were 830 articles remaining for title and abstract screening. 786 articles were subsequently excluded for lack of relevance and a further eight were excluded for not having full‐text availability. 36 articles underwent full‐text analysis, and 18 articles remained for final inclusion and data extraction. A summary of our review process is described in the PRISMA diagram (Figure 1), and a summary of all 18 included articles is available in Table 2. The results reported in this review are structured around study characteristics, methodological approaches, and economic parameters.

**TABLE 2:** Key characteristics of included health economic evaluations.

Author, year, country, reference	Population	Intervention	Comparators	Model type	Methods of analysis	Accuracy: sens./spec.	Costs (as described by papers)	Outcomes	Perspective	Time horizon	Adherence to follow‐up	Outlook for AI
Ahmed et al., 2025, USA [19]	Paediatric patients	IDx‐DR, EyeArt, AEYE‐DS	Manual grading (eye care provider [ECP])	Decision model	CEA	AI: 89.3%/88.2% Manual: 33%/95%	Direct Medical AI costs: Acquisition per site ($10 000) IT integration ($3000) Ongoing support ($10 000) Facility space ($2800) Optician productivity/h ($33) Direct Medical ECP costs: ECP screening ($172)	Additional patients screened Additional patients followed up	Healthcare system	12 months	AI: 55%–64% ECP: 29%–95%	Positive Cost‐effective, especially in larger health systems
Karabeg et al., 2024, Norway [20]	Adult patients in Oslo	EyeArt	Manual grading (ophthalmologist)	N/A	CMA	AI: 100%/100% Manual: 100%/100%	Direct Medical AI costs: USA reimbursement code for AI screening ($33) Indirect AI costs: Transport ($73) Patient time: 1 h ($24) Direct Medical ECP costs: ECP screening ($164) Indirect ECP costs: Transport ($73) Patient time: 1.5 h ($36)	N/A	Healthcare system (extended)	Pilot	N/A	Positive Cost‐saving
Wang et al., 2024, China [21]	Adult patients in China	Different AI algorithm accuracies	AI model scenarios: most accurate AI model	Hybrid decision tree‐Markov model	CUA	AI: 93.3%/87.7% Manual: N/A	Direct Medical AI costs: AI screening ($3.52) Indirect AI costs: Transport ($1.45) Lost income ($5.68) Direct Medical ECP costs: Annual salary ($28 999) Follow‐up costs and blindness costs also analysed	QALYs	Societal	30 years	AI: 50% ECP: 50%	Positive Best cost‐effective sensitivity of 96.9% and specificity of 80.3% (96.9%/77.5% for urban and 94.7%/85.6% for rural subgroups)
Hu et al., 2024, Australia [22]	Adult Indigenous & non‐Indigenous patients	Eyetelligence	Manual grading (GP, optometrist, or ophthalmologist)	Decision analytic Markov model	CEA/CUA	AI: 87%/90.7% any DR 97%/91.4% VTDR 95%/92.9% DME Optometrist: 75%/94% any DR 73%/93% ref. DR GP: 43.3%/94% any DR 56%/98% ref. DR Ophthalmologist: 84.8%/95.5% any DR 84.8%/95.5% ref. DR	Direct Medical AI costs: AI screening ($12.20 AUD) Direct Medical ECP costs: Optometrist screening ($70.55 AUD) Ophthalmologist screening ($91.80 AUD) GP screening ($52.85 AUD)	QALYs Incremental VTDR cases Decremental blindness cases	Healthcare system	40 years	AI: 20.9% (Indigenous patients) and 67.1% (non‐Indigenous patients) ECP: 20.9% (Indigenous patients) and 67.1% (non‐Indigenous patients)	Positive Cost‐effective, especially with universal screening program
Li et al., 2023, China [23]	Adult patients in Changzhi city	EyeWisdom	(i) No screening (ii) Manual grading (ophthalmologist)	Decision analytic Markov model	CUA	AI: 90.8%/98.5% Manual: 96%/94.7%	Direct Medical AI costs: AI screening ($24 538 annually) Diagnosis ($84.87/person) Direct Medical ECP costs: Ophthalmologist screening ($34 703 annually) Diagnosis ($84.87/person) Direct non‐medical costs: Transport ($2.86/person)	QALYs	Healthcare system	50 years	AI: 100% ECP: 100%	Positive Cost‐effective, compared with both manual grading and no screening
Srisubat et al., 2023, Thailand [24]	National DR screening program	ARDA	Manual grading (human grader)	Hybrid decision tree‐Markov model	CUA	AI: 95%/98% Manual: 73.7%/98.6%	Direct Medical AI costs: AI screening ($1/person) Direct Medical ECP costs: ECP screening ($1.70/person)	QALYs	Healthcare system and societal	Lifetime	AI: 60% ECP: 60%	Positive Cost‐effective
Lin et al., 2023, China [25]	Shanghai	EyeWisdom	Manual grading (retinal expert)	Decision analytic Markov model	CEA/CUA	AI: 80.5%/98% Manual: 100%/100%	Direct Medical AI costs: AI screening ($9.60) Diagnosis ($57) Direct Medical ECP costs: Telehealth screening ($10.10) Diagnosis ($57)	QALYs Years without blindness	Societal	30 years	AI: 50.4% ECP: 50.4% +/−25% for sensitivity analysis	Negative Not cost‐effective, compared with telehealth screening
Liu et al., 2023, China [26]	Rural and urban China	ARIAS (specific algorithm unspecified)	(i) No screening (ii) Manual grading (ophthalmologist) (iii) Manual grading (professional grader)	Decision analytic Markov model	CUA	AI: 91.8%/90% non‐STDR 91.8%/90% STDR 59%/90% DME Manual telemed: 91%/97% non‐STDR 91%/97% STDR 59%/97% DME Manual non‐telemed: 76%/98% non‐STDR 95%/98% STDR 82%/98% DME	Direct Medical AI costs: AI screening ($6) Diagnosis ($265) Direct Medical ECP costs (telehealth): Telehealth screening ($11) Diagnosis ($265) Direct Medical ECP costs: Screening ($21) Diagnosis ($265)	QALYs Years without blindness	Societal	30 years	AI: 19% (rural) and 57% (urban) ECP: 19% (rural) and 57% (urban)	Positive Cost‐effective, however also included glaucoma, AMD, myopia, cataracts screening collectively
Gomez Rossi et al., 2022, Brazil [27]	Brazil	IDx‐DR	Manual grading (ophthalmologist)	Decision analytic Markov model	CUA	AI: 87.2%/90.2% Manual: 82.9%/90.3% [28]	Direct Medical AI costs: AI diagnosis ($559) Direct ECP costs: ECP diagnosis ($533)	QALYs	Brazilian taxpayer	Lifetime	Not discussed	Neutral Cost‐effectiveness depends on fee for service and treatment outcomes
Huang et al., 2022, China [29]	Rural China	Airdoc	Manual grading (ophthalmologist)	Hybrid decision tree‐Markov model	CUA	AI: 90.8%/98.5% Manual: 96%/96.5%	Direct Medical AI costs: AI screening ($1.45) Eye exam ($1.63) Direct ECP costs: ECP screening ($3.21) Eye exam ($1.63) Direct non‐medical costs: Screening transport ($0.58) Indirect Costs: Screening income loss ($3.18)	QALYs	Healthcare system and societal	35 years	AI: 100% ECP: 100%	Positive Cost‐effective
Fuller et al., 2022, USA [30]	Low‐income patients	EyeArt	Manual grading (eye care provider [ECP])	Hybrid decision tree‐Markov model	CUA	AI: 100%/65.7% Manual: Not explicitly stated	Direct Medical AI costs: AI screening ($31) Direct ECP costs: Not explicitly stated	QALYs	Payor	5 years	AI: 54.9% ECP: 11.5%	Positive Cost‐effective
Xie et al., 2021, USA [31]	Singaporean national DR screening program	Singaporean DLS	Manual grading (human grader)	Decision tree model	CMA	Autonomous AI: 89.9%/81.8% Semi‐autonomous AI: 89.9%/99.6% Manual: 89.9%/99.2%	Direct Medical AI costs: Fully automated screening ($66) Semi‐automated screening ($62) Direct ECP costs: ECP screening ($77)	N/A	Healthcare system	12 months	Not discussed	Positive Cost‐saving
Chen et al., 2020, Singapore [32]	Private practices	IDx‐DR	Model: AI fees & Medicare reimbursement	N/A	Marginal revenue analysis	AI: 87%/91% Manual: N/A	Direct Medical AI costs: AI screening ($25) AI purchasing plan (up to $13 000)	Marginal revenue per patient	Healthcare providers	12 months	Not discussed	Negative Current financial models not conducive to adoption
Wolf et al., 2020, USA [33]	Paediatric patients	IDx‐DR	Manual grading (eye care provider [ECP])	Decision tree model	CEA	AI: 87.2%/90.2% Manual: N/A	Direct Medical AI costs: AI screening ($0–$100) Direct Medical ECP costs: ECP screening ($35–$500)	True positive proportion	Patient and family	12 months	Adherence to screening influenced conclusions	Neutral Cost‐effective, if adherence to ECP screening > 23% (base case: 20%)
Tufail et al., 2017, UK [34]	Homerton University Hospital, London	iGradingM, Retmaker, EyeArt	Manual grading (human grader)	Decision ree model	CEA	EyeArt AI: 94.7%/20% any DR 93.8%/— ref. DR 99.6%/— PDR Retmaker AI: 73%/52.3% any DR 85%/— ref. DR 97.9%/— PDR Manual: N/A	Direct Medical AI costs: EyeArt screening ($34.26) EyeArt pre‐screening ($33.30) Retmaker screening ($31) Retmaker pre‐screening ($32.48) Direct Medical ECP costs: ECP screening ($39.25)	Appropriate screening outcome	Healthcare system	17 months	Not discussed	Positive Cost‐effective
Prescott et al., 2014, UK [35]	Seven UK screening centres	UK Automated algorithm for DME	Manual grading (human grader)	Decision analytic Markov model	CUA	AI: 75.9%/73.7% Manual: 72.6%/66.8% England 59.5%/79% Scotland 73.3%/70.9% Hybrid	Direct Medical AI costs: AI screening ($147.17/year) Direct Medical ECP costs: ECP screening Scotland ($139.87/year) ECP screening England ($153.44/year)	QALYs	Healthcare system	20 years	Not discussed	Neutral Inferior to Scotland, superior to England as of 2014
Scotland et al., 2010, Scotland [36]	Three screening centres in Scotland	UK Semi‐Automated algorithm	(i) Earlier version of automated algorithm (ii) Manual grading (human grader)	Decision tree model	CEA/CUA	AI: 79.6%/63.4% mild DR 95%/‐ ref. DR Manual (L1 Grader): 81.9%/92% mild DR 99.2%/‐ ref. DR	Direct Medical AI costs: AI screening ($0.17) Direct Medical ECP costs: ECP screening ($1.91)	QALYs Additional case detected Additional appropriate outcome	Healthcare system	20 years	Not discussed	Positive Cost‐effective
Scotland et al., 2007, Scotland [37]	Grampian region	UK Semi‐Automated algorithm	Manual grading (human grader)	Decision tree model	CEA	AI: 85.9%/67.4% mild DR 98%/‐ ref. DR Manual: 81.9%/92% mild DR 99.2%/‐ ref. DR	Direct Medical AI costs: AI screening ($0.18) Direct Medical ECP costs: ECP screening ($1.91)	Additional appropriate outcome Additional true referable case	Healthcare system	12 months	Not discussed	Positive Likely cost‐effective

### 3.2 Study Characteristics

China was the predominant setting for health economic evaluations of AI‐enabled DR screening with five of the eighteen included studies [21, 23, 25, 26, 29]. Four studies were conducted in the United States [19, 30, 31, 33], four in the United Kingdom (including two in Scotland) [34, 35, 37, 38], and one each in Australia [22], Brazil [27], Norway [20], Singapore [32], and Thailand [24]. The earliest study was published in 2007 [37], with most (*n* = 13) published since 2020, indicating a growing interest and evidence generation in the field of AI‐enabled DR screening.

Commercial AI technologies dominated high‐income country evaluations, with EyeArt (Eyenuk) [19, 20, 30, 34] and IDx‐DR (now LumineticsCore, Digital Diagnostics) [19, 27, 31, 33] most frequently assessed. Chinese researchers primarily utilised regionally developed algorithms including EyeWisdom (Zhiyuan Huitu) [23, 25] and Airdoc [29]. Earlier UK studies [35, 37, 38] examined first‐generation automated grading systems, contrasting with more recent deep learning approaches.

### 3.3 Quality Assessment and Risk of Bias in Studies

**FIGURE 2:**
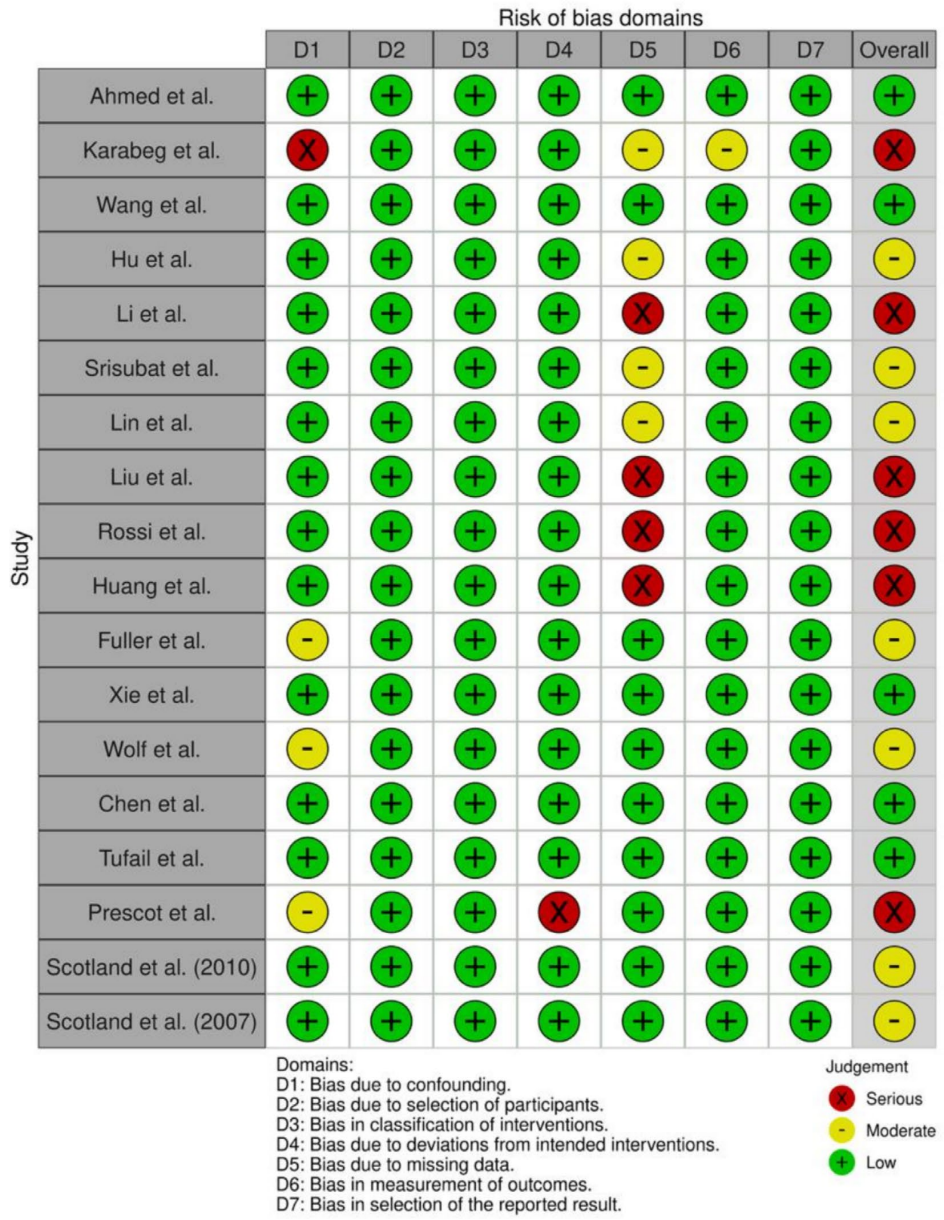
ROBINS‐I risk of bias assessment for included studies.

Our risk of bias assessment using ROBINS‐I (Figure 2) identified several key sources of bias across the included studies. Domain 5 (bias due to missing data) was a key differentiator between studies due to variable consideration of real‐world data on adherence to follow‐up after AI versus manual grading. Studies were assigned serious risk of bias if they did not include this parameter; moderate risk if they included this parameter but assumed equivalence between AI and manual grading; and low risk if they integrated different rates of adherence to follow‐up for AI and manual grading. Additional methodological limitations were identified including widespread reliance on diagnostic accuracy parameters from in silico validation studies rather than real‐world evidence and inadequate consideration of image gradability—both factors that may overestimate AI performance in real‐world settings.

Study design characteristics significantly influence overall bias assessments, with Chen et al.'s marginal revenue analysis and Xie et al.'s cost‐minimisation analysis receiving low risk by exempting effectiveness parameters as an inherent part of their study design. While not biased, these examples demonstrate the need to compare study quality by study design and proceed with caution when comparing results between different study designs, for example, cost‐utility and cost‐minimisation analyses.

Our quality assessment (Table 3) revealed significant methodological limitations across the 18 included studies that affect their reliability and transferability across different healthcare contexts. Most critically, none employed standardised utility measurement approaches for deriving QALYs, instead relying on heterogeneous sources without clear cross‐cultural validation, while approximately one‐third reported financial conflicts of interest with AI companies through employment, patent ownership, or direct financial relationships. The transferability of findings is substantially constrained by diverse healthcare contexts across low‐, middle‐, and high‐income countries, each with distinct cost structures, disease prevalence patterns, and healthcare delivery models. Healthcare decision‐makers should recognise the need for context‐specific economic evaluations that reflect local healthcare delivery models, epidemiological patterns, and economic conditions.

**TABLE 3:** NICE quality assessment of included articles.

Author (reference)	Study limitations	Comments
Ahmed et al. [19]	Potentially serious limitations	Short time horizon (1 year); outcomes are operational versus clinical
Karabeg et al. [20]	Very serious limitations	Very small sample size and lack of generalisability
Wang et al. [21]	Minor limitations	Some reliance on resource use assumptions
Hu et al. [22]	Minor limitations	Some transition probabilities informed by international data
Li et al. [23]	Potentially serious limitations	Some methodological assumptions (e.g., 100% adherence), and some concerns about parameter sources
Srisubat et al. [24]	Potentially serious limitations	High quality economic evaluation. Limitations relate to some parameters relying on assumptions and several authors have financial conflicts of interest with Google (developer of AI technology).
Lin et al. [25]	Minor limitations	The assumption of 100% accuracy for manual grading is the main limitation, though partially addressed through sensitivity analysis.
Liu et al. [26]	Minor limitations	Inherent limitations regarding generalisability of findings.
Gomez Rossi et al. [27]	Potentially serious limitations	Limitations due to costings and assumptions about adherence to DR follow‐up
Huang et al. [29]	Minor limitations	Inherent limitations regarding generalisability of findings.
Fuller et al. [30]	Minor limitations	Main limitations are 5‐year time horizon (vs. lifetime) and exclusion of indirect costs
Chen et al. [32]	Not applicable	Study limited by its design as a focused economic analysis of financial barriers to implementation.
Xie et al. [31]	Potentially serious limitations	Patent conflicts of interest and exclusion of longer‐term health outcomes and treatment costs
Wolf et al. [33]	Potentially serious limitations	Conflict of interest, limited time horizon, patient‐only cost perspective, and US‐specific context
Tufail et al. [34]	Minor limitations	Minor limitations relate to short time horizon and focus on screening rather than health outcomes
Prescott et al. [35]	Minor limitations	Minor limitations relate to utility measurement approach and potential conflicts regarding automated grading
Scotland et al. [36]	Minor limitations	Minor limitations include focus on single screening cycle in primary analysis and potential implementation‐related conflicts of interest
Scotland et al. [37]	Potentially serious limitations	Short time horizon, focus on screening rather than health outcomes, and implementation‐related conflicts of interest

### 3.4 Types of Economic Analyses and Modelling

Cost‐utility analysis (CUA) was the most common health economic approach used by eleven studies (61%) [21, 22, 23, 24, 25, 26, 27, 29, 30, 35, 38], followed by cost‐effectiveness analysis (CEA) used by seven studies (39%) [14, 17, 20, 27, 29, 31, 32]. Of note, three studies utilised both CUA and CEA methods [17, 20, 32]. The methodological approaches used across the included studies reveal significant patterns that warrant further examination.

Singapore as a high‐income country conducted a cost‐minimisation analysis demonstrating a focus on cost differences between AI and the status quo, assuming equivalent effectiveness and screening access between automated, semi‐automated, and standard models of care [31]. Tailored methodological approaches like those used in the USA reflect a diversity of payors for AI‐enabled DR screening with a mix of private insurance, public programs (Medicare for elderly, Medicaid for low‐income), and out‐of‐pocket payments [39]. The USA studies reflect this diversity, with Fuller et al. [30] specifically examining low‐income populations, Chen et al. [32] focusing on a business case analysis using hypothetical billing codes, while Wolf et al. [33] and Ahmed et al. [19] considered paediatric populations who might be covered under various insurance arrangements. These methodological differences reflect an array of healthcare system priorities between and within countries, and demonstrate the importance of context‐specific methodological approaches to health economic analyses of AI‐enabled DR screening.

The modelling approaches used by studies aligned with their time horizons and complexity of the analyses. Markov modelling (*n* = 10) [21, 22, 23, 24, 25, 26, 27, 29, 30, 35], was used in studies with longer time horizons (20+ years) and was the primary method for CUAs. All but one Markov model used annual cycles in keeping with most DR screening interval recommendations [40]. Markov models are particularly suitable for modelling chronic progressive conditions like DR, where patients transition between different health states over time [41]. Decision tree modelling (*n* = 8) [21, 24, 29, 30, 32, 33, 34, 38] was typically used for shorter‐term analyses and CEAs or CMAs. Four studies used a hybrid decision tree–Markov model approach, which combined both methods [21, 24, 29, 30].

Across the included studies, the data used to estimate health economic parameters for AI‐enabled DR screening spanned from 2000 to 2015, with transition probabilities often derived from sources outside the study's country of origin. For example, a Chinese study by Li et al. [23] utilised transition probabilities from Tung et al. [42], a study conducted in Taiwan, rather than mainland China‐specific data. Furthermore, the Australian study by Hu et al. [22] adapted transition probabilities from multiple international sources rather than Australian‐specific epidemiological data. Specifically, they utilised transition probabilities derived from Lian et al. [43] (Hong Kong), Vijan et al. [44] (US), McCarty et al. [45] (Australia), Royle et al. [46] (UK), and Aoki et al. [47] (US) for their Markov model. This distribution reflects varying availability of context‐specific economic parameters, with many studies using international rather than local epidemiologic data, potentially limiting generalisability to local populations.

### 3.5 Interventions and Cost Parameters

#### 3.5.1 Screening Costs and Technologies

Screening costs varied substantially across settings, influenced primarily by the specific AI technologies deployed and their implementation models. Technology acquisition and implementation costs for commercial AI systems vary and may depend on institutional arrangements; however volume‐based, subscription and upfront acquisition finance models are available. Commercial systems with regulatory approval dominated high‐income settings, with EyeArt [19, 20, 30, 34] and IDx‐DR [19, 27, 31, 33] being most prevalent. These established systems typically involved higher licensing fees but offered standardised implementation pathways. In contrast, Chinese studies often evaluated locally developed algorithms such as EyeWisdom [23, 25] and Airdoc [29] potentially reflecting both market access considerations and cost constraints. A critical observation is that most studies inadequately detailed the actual pricing structures of AI technologies, with licensing models ranging from per‐patient fees (most common in US studies) to bulk institutional purchases (prevalent in Asian studies), making direct cost comparisons challenging. Studies also rarely accounted for potentially declining costs of AI technologies over time; therefore perhaps overestimating long‐term expenses.

#### 3.5.2 Direct Medical Costs

Direct medical costs beyond screening demonstrated more consistency across studies, typically including ophthalmology consultations, diagnostic procedures (OCT, fluorescein angiography), and treatments (laser photocoagulation, anti‐VEGF injections, vitrectomy). The cost of manual grading emerged as a key differentiating factor between high and middle‐income settings. Labour costs for ophthalmologists and trained graders were substantial drivers in high‐income settings like Australia [22], the UK [34, 37, 38] and Singapore [32], where AI demonstrably reduced personnel expenses. In contrast, upper‐middle‐income settings (China, Thailand, Brazil) reported relatively modest manual grading costs, diminishing this particular advantage of AI implementation.

Treatment costs for advanced disease states showed remarkable consistency across studies regardless of setting, and this emerged as one of the most significant cost drivers for longer‐term analyses. For example, the cost of anti‐VEGF therapy ($256–$350 AUD per injection) [22] and vitrectomy ($1592–$1947 AUD) [22], dwarfed all screening‐related expenses and highlights the importance of appropriate follow‐up among patients identified with referable DR. The benefits of early detection through routine screening are contingent on patients subsequently accessing the required care through follow‐up. Importantly, a lack of evidence regarding patient adherence to follow‐up after AI‐enabled DR screening limits the comprehensiveness of health economic evaluations for estimating differences in downstream costs and benefits.

#### 3.5.3 Direct Non‐Medical and Indirect Costs

Transportation costs emerged as significant direct non‐medical costs in studies from rural settings, where geographic barriers to care substantially impacted the cost‐effectiveness of interventions. Chinese rural studies [23, 26, 29] considered patient travel expenses and accommodation for hospital visits as direct non‐medical costs, and productivity losses from time away from work as indirect costs. These non‐medical and indirect costs often approximated the direct medical costs of screening itself in rural areas, fundamentally altering the cost‐effectiveness equation compared to urban settings.

### 3.6 Diagnostic Accuracy and Cost‐Effectiveness Implications

Across the 18 studies analysed, we observed substantial variation in reported AI diagnostic accuracy that directly influenced cost‐effectiveness outcomes. As shown in Table 2, sensitivity ranged from 75.9% to 100% and specificity from 20% to 100% across AI systems, demonstrating the wide spectrum of performance capabilities. Notably, the relationship between diagnostic accuracy and cost‐effectiveness was not linear. Wang et al. [21] explicitly modelled this relationship, finding that the algorithm with optimal cost‐effectiveness in China (sensitivity 96.9%, specificity 80.3%) differed from the technically most accurate algorithm (sensitivity 93.3%, specificity 87.7%). This pattern was particularly pronounced in rural settings, where studies by Li et al. [23], Liu et al. [26], and Huang et al. [29] demonstrated that transportation costs and access barriers amplified the economic impact of false positive results.

### 3.7 Study Perspective and Time Horizon

The perspective of health economic evaluations varied by country and healthcare system structure. Nine studies (50%) used a healthcare system perspective, considering only costs borne by the healthcare provider or payer. Five studies (28%) adopted a societal perspective, incorporating direct medical costs alongside direct non‐medical costs such as transportation, and indirect costs including patient time and productivity losses. These societal perspective analyses [21, 24, 25, 26, 29] captured the broader economic impacts of DR screening and consistently demonstrated more favourable cost‐effectiveness ratios for AI implementation in rural regions. Studies focusing solely on healthcare system perspectives [19, 32] necessarily omitted these considerations, potentially underestimating the full economic value of AI screening in underserved areas.

Time horizons ranged from 12 months to lifetime. Seven studies (39%) used short‐term horizons of less than 2 years, primarily for decision tree models or pilot implementations. Ten studies (56%) used long‐term horizons of 20 years or more, typically with Markov models to capture the progression of DR and its complications over a patient's lifetime. The choice of time horizon significantly impacted the types of outcomes measured, with longer horizons more likely to capture vision‐threatening complications and blindness due to non‐diagnosis, or non‐adherence to treatment including follow‐up.

### 3.8 Implementation and Adherence Considerations

There is emerging evidence to suggest the use of AI improves patient adherence to ophthalmology follow‐up after DR screening compared with manual grading, among those with referable disease [48, 49, 50]. This is attributed to the immediate feedback of AI which enables counselling and referral at the point of care, in contrast to remote grading by clinicians [50].

Very few health economic evaluations incorporated adherence to follow‐up as a parameter in their analyses. Only two studies in the USA by Ahmed et al. [19] and Fuller et al. [30] explicitly modelled differences in patient adherence to follow‐up between AI and manual screening pathways. This additional parameter was informed by real‐world evidence collected through a clinical study by Liu et al. [48] contextualised to North America. Fuller et al. [30] accounted for improved patient adherence to follow‐up among those screened with AI versus manual grading, whereas Ahmed et al. [15] accounted for varying ranges of adherence to follow‐up among AI versus manual grading. Regardless, both studies acknowledged the potential implications of follow‐up parameters on cost‐effectiveness.

Most studies modelled for equivalent adherence to follow‐up recommendations between AI and manual grading, which may not reflect real‐world implementation challenges. This parameter choice potentially underestimates the value of AI screening given the evidence suggesting improved patient adherence with AI‐enabled pathways compared to manual grading. Indeed, Lin et al. [25] applied a wider sensitivity range of ±25% to rates of ophthalmology follow‐up due to limited evidence, suggesting adherence to referrals was an area of uncertainty warranting further research in specific healthcare settings. Sensitivity analysis is a viable health economic method to capture the range of potential effects AI‐enabled DR screening might have on patient adherence to follow‐up, in the absence of real‐world data.

### 3.9 Reporting Quality and Guidelines Adherence

Of the 18 studies, only 7 (39%) explicitly reported their research using the Consolidated Health Economic Evaluation Reporting Standards (CHEERS) checklist [51] for standardised reporting of cost‐effectiveness studies. None of the studies mentioned AI‐specific reporting guidelines such as CHEERS‐AI [52] or the Population, Intervention, Comparator, Outcome, Timing, Setting, Communication, Technology, and Context (PICOTS‐ComTeC) framework [53] which address the specific characteristics and complexities of digital health interventions. However, these guidelines were published in 2024 which post‐dates most studies in this review.

## 4 Discussion

We have demonstrated a variety of methodological approaches and economic parameters for health economic evaluations of AI‐enabled DR screening. Our discussion will consider several key findings from our analysis, including context‐specific parameter selection, assumptions about patient adherence to follow‐up after AI‐enabled DR screening, and the trade‐off between diagnostic accuracy and cost‐effectiveness. We will also retrospectively apply concepts from new health economic reporting guidelines, CHEERS‐AI and PICOTS‐ComTeC, noting the underutilisation of reporting guidelines among included studies.

### 4.1 Parameter Selection

Contextualised parameters are essential for accurate health economic modelling. We noted significant variation in parameter values between countries and, in some instances, within countries. Labour costs associated with manual grading, for example, were much greater in high‐income countries such as Australia [22], the United Kingdom [34, 35, 37, 38] and Singapore [32]. AI‐enabled DR screening was highly cost‐effective in these settings, largely due to reduced labour costs associated with automated grading. In contrast, countries such as China as an upper‐middle income country with lower labour costs experienced a smaller change in costs of grading between AI and manual graders [21, 23, 25, 26, 29]. Conclusions about cost‐effectiveness in one country have limited generalisability to other countries due to variation in economic systems.

Cost‐effectiveness of AI‐enabled DR screening may also vary within the same country and depends on the perspective used. For example, transport costs (a direct non‐medical cost) associated with DR screening were a major determinant of cost‐effectiveness in rural areas of China [23, 29] in contrast to metropolitan areas with more proximal access to services. For patients in geographically isolated areas, the burden of travel to a healthcare setting can be substantial [54] and this should be considered by health economists studying countries with such dispersion of populations, for example, Australia. Cost‐effectiveness analyses conducted using non‐generalisable data from another setting, such as grouping metropolitan and rural populations together, may jeopardise the validity of their results. We recommend researchers conduct separate analyses for rural and metropolitan populations or incorporate an appropriate range of travel costs into sensitivity analyses.

QALYs were the preferred measure of effect across studies, particularly in upper‐middle‐income countries such as China [21, 23, 25, 26, 29], Thailand [24] and Brazil [27] which almost uniformly conducted a CUA. This trend may reflect a priority to expand screening capacity and therefore demonstrate changes in effectiveness, as well as costs associated, with AI screening. Thus, a universal metric of cost per QALY was warranted for comparison with other public health interventions competing for funding. Natural measures of effect used in CEAs such as years without blindness [25, 26], additional cases detected [38], or additional appropriate outcomes [34, 37, 38] may be valuable in settings where a screening program with manual grading is already established. Appropriate comparison can therefore be made between DR‐related effects of the status quo versus AI screening, in contrast to generic effects, that is, QALYs for comparison with non‐DR conditions.

### 4.2 Patient Adherence to Follow‐Up

The clinical effectiveness of AI‐enabled DR screening is dependent on patient adherence to follow‐up recommendations. Emerging evidence suggests improved patient adherence to ophthalmology review after undergoing screening with AI compared with manual grading [48, 49, 50]. The theory underpinning improved adherence to follow‐up is linked with immediate feedback and opportunistic counselling for patients screened by AI [49].

Most studies in our review modelled for equivalent adherence to follow‐up [21, 22, 23, 24, 25, 26, 29], or did not include adherence to follow‐up as a parameter [20, 27, 32, 33, 34, 35, 37, 38], and this may result in an underestimation of the true cost‐effectiveness of AI‐enabled DR screening. Most studies which did not include adherence to follow‐up as a parameter were earlier evaluations prior to 2023 or had shorter time horizons of less than 5 years. Further real‐world implementation research should be conducted in specific healthcare contexts to inform future cost‐effectiveness studies which integrate data relating to patient adherence to AI recommendations. This is particularly relevant for modelling studies over a prolonged period of time which capture long‐term effects on vision for unmanaged patients, that is, those who do not attend recommended follow‐up.

### 4.3 Diagnostic Accuracy and Cost‐Effectiveness Trade‐Off

The trade‐off between AI sensitivity and specificity represents a critical consideration often overlooked in implementation decisions [21, 55]. While algorithms with high sensitivity minimise undiagnosed DR cases that could progress to blindness and incur substantial treatment costs, high specificity reduces unnecessary referrals—particularly valuable in rural settings where patients face significant travel costs and lost wages [54, 56]. Our review identified Wang et al.'s study [21] as the only article to explicitly model this relationship through 1100 different diagnostic performance scenarios, revealing that the most cost‐effective algorithm for China (sensitivity 96.9%, specificity 80.3%) was not the technically most accurate one (sensitivity 93.3%, specificity 87.7%). Their findings challenge the assumption that maximising overall accuracy should be the primary implementation goal.

Commercial AI systems demonstrate significant variation in their inherent sensitivity‐specificity balance, as highlighted by Tufail et al. [34], who found substantial differences in how technologies like Retmaker and EyeArt performed with respect to these metrics. Such variability means the theoretically optimal operating point identified through modelling may not be achievable with available commercial systems. Local implementation contexts further complicate this balance, as the economic consequences of false positive and false negative results vary between healthcare settings. Policymakers must therefore juggle competing priorities—maximising case detection while minimising unnecessary referrals—with decisions tailored to local healthcare constraints, disease prevalence, and patient barriers.

Future economic evaluations should adopt more comprehensive approaches that incorporate real‐world AUROC curves of commercially available technologies rather than theoretical performance parameters. Models should be contextualised to local disease prevalence patterns and include detailed analysis of the differential costs associated with false positive and false negative results in specific implementation contexts. This approach would provide more realistic assessments of how AI screening systems will perform economically when deployed at scale, acknowledging that a universal “optimal” operating point does not exist across all settings and technologies. Ultimately, the goal is to identify the most appropriate sensitivity‐specificity balance for each unique healthcare context rather than pursuing maximum technical accuracy.

### 4.4 Application of Updated Reporting Guidelines for Health Economic Evaluations

Retrospectively applying the CHEERS‐AI [52] framework revealed several methodological gaps in existing economic evaluations. Most studies inadequately describe the integration of AI systems with existing clinical workflows and IT infrastructure, potentially underestimating true implementation costs that extend well beyond software acquisition. Additionally, few studies explicitly model AI system failures, such as algorithm errors, technical outages, or poor‐quality image inputs requiring repeated retinal imaging or clinician review, and their associated economic consequences. These omissions represent missed opportunities for more realistic cost‐effectiveness modelling as the economic impact of maintaining systems, and managing equivocal results can substantially affect real‐world cost‐effectiveness.

When viewed through the PICOTS‐ComTeC lens [53], existing evaluations demonstrate inconsistencies in reporting interoperability requirements, thereby excluding potentially significant costs for system integration with electronic health records and imaging platforms. Most studies inadequately assess implementation factors, such as IT capability, staff technical literacy, and governance frameworks, which heavily influence implementation success and therefore real‐world cost‐effectiveness. Furthermore, setting‐specific adaptation requirements including training needs, ongoing technical support, and cultural factors affecting technology acceptance are typically underreported, despite their substantial impact on implementation costs and long‐term sustainability. These shortcomings highlight the need for more comprehensive evaluation frameworks tailored to the unique implementation challenges of healthcare AI technologies.

### 4.5 Strengths and Limitations

Our review identifies several knowledge gaps in existing health economic evaluations of AI‐enabled DR screening which are important for clinical implementation. Our analysis of 18 studies across diverse settings demonstrates key health economic considerations including the cost‐effectiveness trade‐off between sensitivity and specificity, context‐specific parameter selection reflecting local healthcare constraints, and differential patient adherence to ophthalmological follow‐up between AI and manual screening pathways. We emphasise the incorporation of real‐world evidence in future and updated economic evaluations, combined with recommendations for specialised reporting frameworks such as CHEERS‐AI and PICOTS‐ComTeC. Our contemporary appraisal calls for future studies to address these limitations which recognise the inherent complexities of AI‐enabled DR screening and improve the clinical utility of economic analyses.

This review has several limitations. Our selection of databases did not include the NHS Economic Evaluation Database (not updated since 2015) and Health Economic Evaluations Database. Exclusion of these databases may have limited the inclusion of all articles of relevance; however our extensive search across the key clinical databases (Embase, MEDLINE and Scopus databases) means we were likely to have identified the majority of recent economic evaluations alongside the screening of DR. We did not compare algorithms that provide diagnostic support across multiple eye conditions including glaucoma and macular degeneration in addition to DR. Such multi‐disease platforms potentially offer greater economic value through shared infrastructure costs but introduce additional complexity in evaluation. AI‐enabled screening for multiple conditions would encounter similar challenges and considerations outlined in this paper, such as implications for accuracy, context‐specific parameters, and adherence to follow‐up on cost‐effectiveness. Also, while the identified diagnostic accuracy and cost‐effectiveness trade‐off presents an intriguing conceptual framework, it primarily draws from a single study [21], limiting its generalisability across diverse healthcare systems and populations.

Our analysis was unable to explore the critical intersection between regulatory requirements and reimbursement policies—factors which significantly influence implementation feasibility and economic sustainability of AI‐enabled DR screening in different countries. There is a dearth of information regarding this key consideration among health economic studies of this technology and it should be considered in future evaluations. Our review also offers limited examination of how different AI business models (subscription‐based, per‐scan pricing, or upfront purchase approaches) might affect long‐term cost‐effectiveness as technologies mature and evolve. Future reviews could address these gaps through comparative analyses incorporating diverse regulatory environments and business models, and health economic studies should consider these less visible costs in their evaluation.

## 5 Conclusion

AI is an accurate tool for the screening of DR, with comparable sensitivity and specificity to specialist ophthalmologists. It is a potentially effective intervention which may address the shortage of screening services in low‐resource areas; however cost‐effectiveness is critical for its widespread implementation. This review identified 18 studies which evaluated the cost‐effectiveness of AI‐enabled DR screening with variation in methodological approaches and parameter selection. Major health economic considerations for the implementation of AI‐enabled DR screening include the trade‐off between diagnostic accuracy and cost‐effectiveness, the necessity of context‐specific parameters that reflect local healthcare systems, and variation in adherence to follow‐up among patients who undergo AI versus manual screening. The methodological rigour of these health economic studies could be improved through the adoption of specialised frameworks designed for digital health interventions. While standard economic evaluation guidelines provide a good foundation for reporting, newly developed frameworks such as CHEERS‐AI and PICOTS‐ComTeC offer additional dimensions crucial for AI evaluation.

## Conflicts of Interest

The authors declare no conflicts of interest.

## Supporting information


**TABLE S1:** Information sources & advanced search query syntax by components.
**TABLE S2:** Eligibility criteria for included studies.
**TABLE S3:** Definitions of health economic analyses.

## Data Availability

Data sharing not applicable to this article as no datasets were generated or analysed during the current study.
