# Supplementary material for: Health Economic Considerations for the Implementation of Artificial Intelligence‐Enabled Diabetic Retinopathy Screening: A Review
Source: Clin Exp Ophthalmol. 2025 Nov 3;54(1):144–61. doi: 10.1111/ceo.70016 (PMC12886596; doi:10.1111/ceo.70016)
Supplement: Supplementary file 1 — TABLE S1: Information sources & advanced search query syntax by components. TABLE S2: Eligibility criteria for included studies. TABLE S3: Definitions of health economic analyses. [file CEO-54-144-s001.docx]

**Supplementary Table 1:** Information Sources & Advanced Search Query Syntax by Components

|  |  | | **Database Search Syntax** | |
| --- | --- | --- | --- | --- |
| **Concept** | **Medline – 433** | **Embase – 195** | | **Scopus – 487** |
| **Artificial Intelligence** | **Search 1** – Artificial Intelligence/ or Neural Networks, Computer/ or exp Machine Learning/ or Image Processing, Computer-Assisted/    **Search 2** – (Automated or "autonomous AI" or "computer based analysis" or "convolutional neural network").ti,ab,kw. | **Search 1** – Artificial intelligence/ or machine learning/ or deep learning/ or convolutional neural network/    **Search 2** – (“Artificial intelligence” or automated or “autonomous AI” or “computer based analysis”).ti,ab,kw.. | | **Search 1 -** INDEXTERMS("Artificial Intelligence") or INDEXTERMS("Neural Networks, Computer”) or INDEXTERMS("Machine Learning") or INDEXTERMS("Image Processing, Computer-Assisted")  **Search 2 -** TITLE-ABS(automated) or TITLE-ABS("autonomous AI") or TITLE-ABS("computer based analysis") or TITLE-ABS("convolutional neural network") |
|  | AND | AND | | AND |
| **Diabetic Retinopathy** | **Search 1** – Ophthalmology/ or Diabetic Retinopathy/    **Search 2** – ("Diabetic eye*" or "diabetic retinopathy" or "retina" or "retinal" or "retinopathy").ti,ab,kw. | **Search 1** – Exp diabetic retinopathy/ or diabetic macular edema/ or ophthalmology/    **Search 2** – (“Diabetic retinopathy” or “diabetic eye disease” or “retina” or “retinal” or “retinopathy” or “diabetic macular ?edema”).ti,ab,kw. | | **Search 1 –** INDEXTERMS(“Diabetic Retinopathy”)  **Search 2 -** TITLE-ABS(“diabetic eye*”) or TITLE-ABS(“diabetic retinopathy”) or TITLE-ABS(“more than mild DR”) |
|  | AND | AND | | AND |
| **Cost-Effectiveness** | **Search 1** – Cost-Benefit Analysis/ or Cost-Effectiveness Analysis/    **Search 2** – (Cost* or "health economic*").ti,ab,kw. | **Search 1** – Health economics/ or cost effectiveness analysis/ or cost benefit analysis/ or cost minimization analysis/ or cost utility analysis/    **Search 2** – (Cost adj2 analys?s.)ti,ab,kw or “health economic*”.ti,ab,kw. | | **Search 1 –** INDEXTERMS("Health economics") OR INDEXTERMS("cost effectiveness analysis") OR INDEXTERMS("cost benefit analysis") OR INDEXTERMS("cost minimization analysis") OR INDEXTERMS("cost utility analysis")  **Search 2 –** TITLE-ABS(Cost* or "health economic*") |

**Supplementary Table 2:** Eligibility Criteria for Included Studies

| Inclusion | Exclusion |
| --- | --- |
| *Population* |  |
| - Patients undergoing DR screening | - Patients undergoing screening for other eye conditions e.g. AMD, glaucoma not including DR |
| *Intervention* |  |
| - AI-supported grading of retinal image including automated and semi-automated screening | - No AI-supported grading of DR - Studies with non-image-based applications of AI relating to DR |
| *Comparator* |  |
| - Manual grading of retinal image in-person or via telehealth (synchronous or asynchronous) | - Studies exclusively comparing AI algorithms with other AI algorithms for detecting DR |
| *Outcome* |  |
| - Standardised measure of cost-effectiveness e.g. incremental cost-effectiveness ratio, cost/unit of effect, cost saving | - Studies which do not report methodology for health economic analysis or a cost-effectiveness outcome |
| *Study Characteristics* |  |
| - Cost-utility analyses - Cost-effectiveness analyses - Cost-minimisation analyses - Cost-benefit analyses - Cost-consequence analyses - Other primary health economic analyses | - Review articles - Non-original research e.g., commentaries, conference abstracts, matters arising - Articles without full-text availability - Articles not written in English |

**Supplementary Table 3:** Definitions of health economic analyses

| **Health economic analysis approach** | **Description of approach** |
| --- | --- |
| **Cost-effectiveness analysis (CEA)**: | A comparative economic method that evaluates alternatives by examining their relative costs and outcomes, where outcomes are measured in a single natural unit (like life-years gained, disease cases prevented, etc.). |
| **Cost-utility analysis (CUA)**: | A specialised form of cost-effectiveness analysis that compares alternatives by measuring outcomes with a generic health status metric that captures both mortality and morbidity effects (typically quality-adjusted life-years [QALYs] or disability-adjusted life-years [DALYs]). |
| **Cost-benefit analysis (CBA)**: | A comparative economic method that evaluates alternatives by expressing both costs and outcomes in monetary terms. This approach values interventions based on affected individuals' preferences, typically measured as willingness to pay. |
| **Cost-minimisation analysis (CMA)**: | A comparative economic method that focuses solely on comparing costs between alternatives that are assumed to produce equivalent health outcomes. |
| **Cost-consequence analysis (CCA)**: | A comparative economic method that evaluates alternatives by presenting their relative costs and multiple outcomes of interest separately, without converting outcomes to a single measure. |
